# The immune mechanism of *Mycoplasma hyopneumoniae* 168 vaccine strain through dendritic cells

**DOI:** 10.1186/s12917-017-1194-1

**Published:** 2017-09-15

**Authors:** Yumeng Shen, Weiwei Hu, Yanna Wei, Zhixin Feng, Qian Yang

**Affiliations:** 1 Veterinary College, Nanjing Agricultural University, Weigang 1, Nanjing, Jiangsu 210095 People’s Republic of China; 2 Institute of Veterinary Medicine, Jiangsu Academy of Agricultural Sciences, Key Laboratory of Veterinary Biological Engineering and Technology, Ministry of Agriculture, National Center for Engineering Research of Veterinary Bio-products, Nanjing, 210014 People’s Republic of China

**Keywords:** Vaccine *Mhp*-168 strain, Dendritic cells, Immune protection

## Abstract

**Background:**

*Mycoplasma hyopneumoniae* (*Mhp*) causes porcine enzootic pneumonia, a disease that cause major economic losses in the pig industry. Dendritic cells (DCs), the most effective antigen-presenting cells, are widely distributed beneath respiratory epithelium, DCs uptake and present antigens to T cells, to initiate protective immune responses in different infections. In this study, we investigated the role of porcine DCs in vaccine *Mhp*-168 exposure.

**Results:**

The antigen presenting ability of DCs were improved by vaccine *Mhp*-168 exposure. DCs could activate T-cell proliferation by up-regulating the antigen presenting molecule MHCII expression and co-stimulatory molecule CD80/86. However, the up-regulation of IL-10 and accompany with down-regulation of IFN-γ gene level may account for the limitation of attenuated *Mhp-168* strain use as vaccine alone.

**Conclusion:**

These findings are benefit for exploring the protection mechanisms and the possible limitations of this attenuated *Mhp-168* vaccine.

## Background

*Mycoplasma hyopneumoniae (Mhp)* causes porcine enzootic pneumonia (EP) and causes tremendous economic losses all over the swine industry [1]. *Mhp* infection is difficult to eradicate and it will long term existence in most herds by direct sow-to-pig exposure [2]. *Mhp* infection causes physical damage on the respiratory tract and modulates the host immune response [3], the primary *Mhp* infection often becomes complicated by secondary bacterial and viral infections [4]. *Mhp* infection causes the release of inflammatory cytokines, such as interleukin (IL)-1β, IL-18 both in vivo and in vitro [5]. In healthy animal, the lower respiratory tract (trachea and lung) is essentially sterile, pathogenic microorganisms have the opportunity to colonize and invade the host when the cilia clearance ability is impaired [6]. *Mhp*-derived lipid-associated membrane proteins (LAMPs) result in either necrosis or apoptosis in macrophages and porcine peripheral blood mononuclear cells (PBMCs) in vitro [7]. Accordingly, cilia clearance loss, macrophages and PBMC dysfunction are responsible for immunosuppression and predisposes pigs to secondary infections. Some drugs and antibiotics are effective against *Mhp*,but they can’t completely clear the infection of *Mhp*. In addition, drug resistance and antibiotic residues are important issues. Vaccination is another essential strategy in controlling *Mhp* infection. Some commercially available vaccines are the inactivated whole cells of *Mhp*, however, inactivated vaccine only provide the partly protection for Mycoplasmal pneumonia of swine (MPS) [8]. Shao et al. have identified that the attenuated *Mhp* 168 strain vaccine developed an effective protection against MPS [9]. However, the exact immunologic mechanism of this attenuated vaccine still has not been researched detailedly.

Dendritic cells (DCs), the most professional antigen-presenting cells (APCs) [10], are widely distributed in the mucosal tissue, preparing for monitor the invasion of pathogens [11]. By extending trans-epithelial dendrites, DCs sample incoming pathogens and migrate to the T-cell region of the mucosa-associated lymphoid tissue (MALT) to trigger an effective immune response [12]. The nasal mucosa barrier is a key impediment for *Mhp* up-take and subsequent antigen-specific adaptive immune responses. The interactions between *Mhp* and DCs have not been investigated. Whether DCs result in the generation of beneficial immune responses during attenuated *Mhp* immune process still unclear.

The vaccine *Mhp*-168 has been used in some Chinese farms with good immune-protection against *Mhp*. Our study aimed to gain a better understanding of the immunologic mechanism of this attenuated vaccine. We have characterized the immune responses of porcine DCs after exposure with vaccine *Mhp*-168 and their capacity to induce T cell responses in vitro.

## Methods

### Animals

A total of 20 five weeks old, Yorkshire, Landrace, and Large White cross-bred pigs were bred and maintained under specific pathogen-free conditions (e.g. Transmissible Gastroenteritis Virus, Porcine Epidemic Diarrhoea Virus, Porcine Circovirus Type 2, Porcine Reproductive and Respiratory Syndrome Virus, *Mhp*). All animal experiments were approved by the Institutional Animal Care and Use Committee of Nanjing Agricultural University (Nanjing, China) and followed National Institutes of Health guidelines.

### Reagents

Antibodies PE-conjugated co-stimulatory molecules cluster of differentiation (CD) 80/86 (ab69778), PE-conjugated CD1a(ab25599), FITC-conjugated Workshop Cluster 3a (SWC3a, ab24885) were obtained from Abcam (New Territories, Hong Kong). FITC-conjugated histocompatibility leukocyte Ag II-DR (MHCII, LS-C129867) was obtained from LifeSpan BioSciences (Seattle, WA, USA). PE-conjugated SWC3a (74-22-15A) was obtained from BD Biosciences (USA). Recombinant swine interleukin 4 (IL-4), was bought from BioSource (USA). Recombinant swine granulocyte-macrophage colony-stimulating factor (GM-CSF), was bought from Invitrogen (USA). Lipopolysaccharide (LPS) was bought from Sigma (Japan). RPMI 1640 medium and 4,6-diamidino-2-phenylindole (DAPI) solution were bought from Invitrogen (Grand Island, NY, USA). Fetal bovine serum (FBS) was bought from MULTICell (Canada). SABC-POD (rabbit IgG) Kit and Peroxidase Substrate Kit were bought from BOSTER (Wuhan, China). RNA extraction kit was bought from TianGen Biotech (Beijing, China).

### Mycoplasma pneumonia 168

Vaccine *M. hyopneumoniae strain 168* (vaccine *Mhp*-168) was an attenuated vaccine strain, which cultured from continuous passage of *Mhp* wild strain [13]. The wild strain was completely attenuated by more than 300 passages in KM_2_ media. The 340th passage of M. *hyopneumoniae* 168 strain was confirmed safety for cross-bred pigs and 360th passage of M. *hyopneumoniae* 168 strain could developed the effective protection against MPS [9]. We obtained vaccine *Mhp*-168 from Jiangsu Academy of Agricultural Sciences,Key Laboratory of Veterinary Biological Engineering and Technology and the 360 th passage of M. *hyopneumoniae* 168 strain was used in our experiment. This vaccine *Mhp*-168 was cultured in KM_2_ medium until the beginning of the stationary phase. Then it was collected after centrifugation at 12,000 × g for 20 min and washed twice with PBS.

### Generation of bone marrow-derived DCs (BMDCs)

BMDCs were isolated and cultured using our advanced method [14]. Briefly, bone marrow was extracted from the femurs of 1.5 months old piglets and treated with red blood cell lysing buffer. The bone marrow cells were differentiated into DCs by suspending the cells in complete medium (RPMI 1640 medium supplemented with 10% foetal bovine serum (FBS), 1% penicillin-streptomycin, and 10 ng/ml GM-CSF and IL-4). After 60 h of culture, non-adherent granulocytes were removed gently by discarding the medium, and fresh medium was added. Non-adherent and loosely adherent cells were harvested on day 6 and sub-cultured overnight in complete medium. On day 7, immature DCs were collected for further experiments.

### Exposure of BMDCs to vaccine *Mhp*-168

On day 7 of the culture, immature DCs were collected and seeded into 24-well plates (5 × 10^5^ cells/well). RPMI 1640 medium (negative control), 10 ng/ml of LPS (positive control) or vaccine *Mhp*-168 (40/dendritic cell) were added for 8 h, 12 h and 24 h incubation period respectively. Both the Mock and the stimulated DCs were collected after 24 h for flow cytometry analysis. Both the Mock and the stimulated DCs were collected at 8 h, 12 h and 24 h respectively for total RNA extraction.

### Flow cytometry

In order to determine the modulation of surface markers on DCs treated with LPS or vaccine *Mhp*-168 in particular time point, treated DCs samples were washed with cold PBS twice and stained with fluorescent mAbs specific for swine or human SWC3a, CD80/86, CD1a and MHCII at 4 °C for 0.5 h as per manufacturer’s guidelines. After washed three times with PBS, FACS was used to detect phenotype of DCs.

### Real-time quantitative PCR analysis of the cytokine secretion by DCs

The total RNA of Mock DCs and stimulation DCs were obtained using TRIzol Reagent (Invitrogen) according to the manufacturer’s instructions. The cDNA was generated by reverse transcription using HiScript TM QRT SuperMix for qPCR (Vazyme) according to the manufacturer’s instructions. Gene expression data were collected using 7500 Real-Time PCR System (Applied Biosystems, Hercules, CA) and analyzed by the △△Ct methods. The relative IL-10/IL-12/IFN-γ mRNA was normalized with glyceraldehyde 3-phosphate dehydrogenase (GAPDH). The specific primers were shown in Table 1.

**Table 1:** Primers used for RT-qPCR

Gene	Sequence (5’ to 3’)
IL-10 forwardIL-10 forward	TCTGAGAACAGCTGCATCCAC
IL-10 reverse	CGCCCATCTGGTCCTTCGTT
IL-12 forward	TCAGAAGGCCAAACAAACCCT
IL-12 reverse	GGCAACTCTCATTCGTGGCTA
INF-γ forward	CGCAAAGCCATCAGTGAACTCA
INF-γ reverse	TCTGGCCTTGGAACATAGTCT
GAPDH forward	TCATCATCTCTGCCCCTTCT
GAPDH reverse	GTCATGAGTCCCTCCACGAT

### Mixed leukocyte reaction (MLR) assay

To analyze the capacity of DCs to activate T lymphocytes, Pig was anaesthetized before exsanguinated. Inguinal lymph nodes were collected for lymphocytes separation. Purified T cells were stained with CFSE following the instruction. CFSE-labelled T-lymphocytes (5 × 10^5^) were co-cultured with Mph-DCs or Mock-DCs at a ratio of 1:1; 1:5 (DCs: lymphocytes). Co-cultures were incubated for five days. Finally, the cells were harvested, and the fluorescence intensity of the CFSE was determined by flow cytometry analysis.

### Statistical analysis

Each of the experiments was performed at least three individual times. Data was performed using Statistical Product and Services Solutions (SPSS) package. Statistical difference was determined by one-way ANOVA followed by Dunnett’s test. *P* values < 0.05 were considered statistically significant.

## Results

### Morphology of BMDCs after 6 days culture

**Fig. 1:**
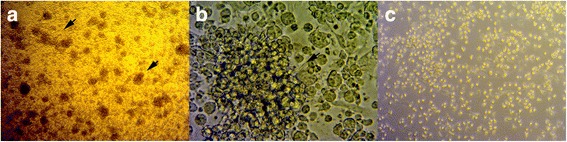
Generation of BMDCs. Porcine BMDCs were cultured in RPMI 1640 medium supplemented with GM-CSF and IL-4. **a** The clusters of BMDCs on day 5, as viewed by optical microscopy at 100 ×magnification. **b** Morphology of cells after 6 days of culture, dendritic processes could be observed using optical microscopy at 400 × magnification. **c** Porcine BMDCs were cultured in RPMI 1640 medium supplemented without GM-CSF and IL-4 as control

Bone marrow-derived cells were cultured for 6 days by using RPMI 1640 media with GM-CSF and rpIL-4 to allow them to differentiate into immature BMDCs. Cell phenotypes were then evaluated by using microscopy. BMDCs formed clusters until the day 5 (Fig. 1a), and dendritic processes could be observed on the day 6 (Fig. 1b). When porcine monocytes were cultured without adding GM-CSF and IL-4, only a few live cells were left and no cell aggregates were observed in the plates by day 6 (Fig. 1c).

### Phenotypic alteration of BMDCs stimulated by vaccine *Mhp*-168 in vitro

**Fig. 2:**
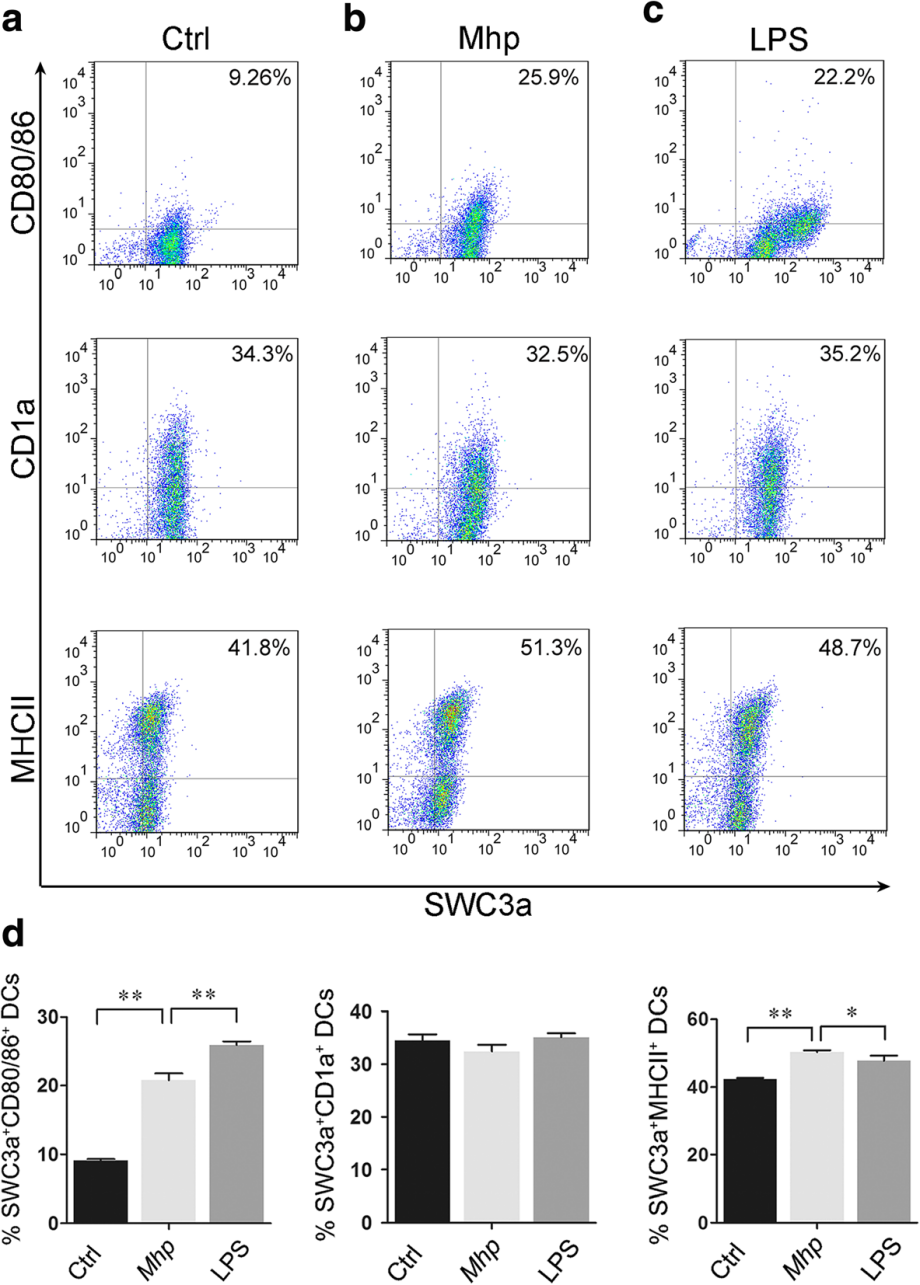
Expression of antigen presenting molecules and co-stimulatory molecules on DCs after vaccine *Mhp*-168 exposure at 24 h.p.i. Immature BMDCs were exposed to vaccine *Mhp*-168 (40/dendritic cell) or LPS (10 ng/ml) as positive control for 24 h. Dot plots show the percentage of CD1a^+^SWC3a^+^DCs, CD80/86^+^SWC3a^+^ DCs and MCHII^+^SWC3a^+^ DCs. **a** Untreated BMDCs. **b** *Mhp* 168 treated DCs. **c** LPS treated DCs. **d** Bar graphs show the percentage of CD1a^+^SWC3a^+^, CD80/86^+^SWC3a^+^, MHCII^+^SWC3a^+^ cells in BMDCs populations after vaccine *Mhp*-168 exposure. Data show the means ± SEM (*n* = 3 per group). All experiments were performed independently three times. Statistical significance was assessed by Student’s *t*-test. Differences were considered significant at (*) 0.01 < *p* < 0.05, (**) *p* < 0.01

DCs phenotypic maturation is essential for T cells activation and immune response. DCs up-regulate the expression of co-stimulatory and antigen presentation associated factors at the mature stage [15]. We used flow cytometry and the antibodies described earlier to detect surface expression of CD80/86, MHCII and CD1a to determine whether vaccine *Mhp*-168 affects the maturation and activation of DCs. By gating on untreated BMDCs, we compared the variation of phenotype between the untreated BMDCs and vaccine *Mhp*-168 treated-BMDCs in the same culture condition. Comparing with untreated immature Mo-DCs, immature Mo-DCs showed changes in surface marker expression characteristic of DC maturation (up-regulation of SWC3a + CD80/86+ and SWC3a + MHCII+), after being stimulated by the vaccine Mhp-168 or LPS (Fig. 2b and c). There is no significant change of SWC3a^+^CD1a^+^ co-stimulatory molecule expression after being exposed to vaccine *Mhp*-168 (Fig. 2a and b). These results suggest that stimulation of immature Mo-DCs triggers maturation.

### Vaccine *Mhp*-168 stimulation modulates the expression of cytokines

**Fig. 3:**
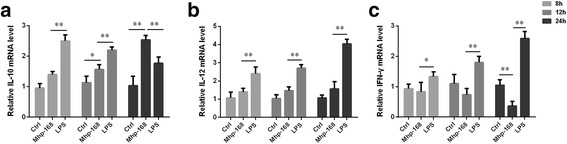
Cytokines mRNA expression levels of DCs after vaccine *Mhp*-168 exposure. **a** IL-10; **b** IL-12; **c** IFN-γ. BMDCs were treated with vaccine *Mhp*-168 or LPS (10 ng/mL) for 8 h, 12 h and 24 h. The mRNA expression levels of IL-10, IL-12, IFN-γ were detected by real-time quantitative PCR (RT-qPCR). Data are expressed as means ± SD (*n* = 3 per group). All experiments were performed independently three times. Statistical significance was assessed by Student’s t-test. Differences were considered significant at (*) 0.01 < *p* < 0.05, (**) *p* < 0.01

Cytokines production by DCs also play an important role in immunomodulation besides the expression of surface molecules [16]. Therefore, we investigated whether vaccine *Mhp*-168 was able to modulate cytokine production by monitoring the mRNA expression level of IL-10, IL-12, INF-γ in vaccine *Mhp*-168 stimulated BMDCs lysis. Immature Mo-DCs stimulated with vaccine *Mhp*-168 show up-regulation of IL-10 and IL-12 mRNA expression level at 8 h incubation and increased at a steady-state level, with maximal production at 24 h incubation (Fig. 3a and b). While down-regulation expression of cytokines was observed in INF-γ at 8 h incubation and at a steady-state level, with maximal production at 24 h incubation (Fig. 3c). All LPS stimulated immature Mo-DCs showed increasing in IL-12, IFN-γ and IL-10 mRNA expression level. These results indicated that immature Mo-DCs have the capacity to activate an immune response to vaccine *Mhp*-168 by up-regulation IL-12 and IL-10 mRNA expression level.

### Vaccine *Mhp*-168 treated-BMDCs promote T cells proliferation

**Fig. 4:**
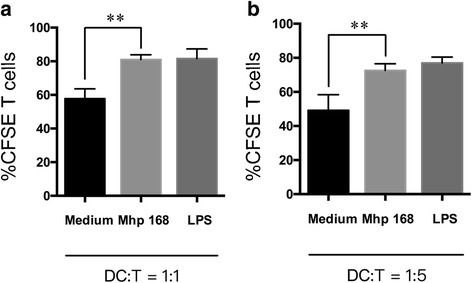
Vaccine *Mhp*-168 treated DCs increased the capacity of T lymphocytes stimulation. Pretreatment DCs with vaccine *Mhp*-168 for 24 h. Vaccine *Mhp*-168-treated DCs were co-cultured with T lymphocytes (ratios 1:1, 1:5) for another 5 days (**a**, **b**). DCs were stimulated with LPS or remained unstimulated then co-cultured with T lymphocytes as positive or control group. T lymphocytes proliferation was evaluated using CFSE. Data are presented as mean ± SEM (*n* = 3 per group). All experiments were performed independently three times. Statistical significance was assessed by Student’s *t*-test. Differences were considered significant at (*) 0.01 < *p* < 0.05, (**) *p* < 0.01

The immune stimulatory properties of DCs to T cells are essential for adaptive immune responses. Here, allogenic mixed lymphocyte reaction (MLR) was performed to determine whether the ability of BMDCs induced T cells proliferation have changed after being treated with vaccine *Mhp*-168. As shown in Fig. 4, mature BMDCs stimulated by LPS up-regulate the ability of T cells proliferation at a DC/T cell ratio of 1:1 and 1:5. The proliferation capacity of T cells were promoted after being stimulated by *Mhp-168* both at a DC/T cell ratio of 1:1 and 1:5.

## Discussion

*Mhp* is the primary etiological agent responsible for swine enzootic pneumonia (EP), which cause tremendous economic losses all over the swine industry [17]. There are many strategies to prevent *Mycoplasma* infection, such as drugs and antibiotics. However, they have not been proved effective inhibition of this pathogens in vivo, and still have problems of resistance and antibiotics residue [18]. Until now, vaccination is one of the essential strategy in controlling EP. Clinical study shows vaccine *Mhp*-168 activated the systemic cellular immunity and local mucosal immunity, which is benefit for prevention and control of EP [9]. Dendritic cells (DCs), the most effective antigen-presenting cells, are widely distributed beneath respiratory epithelium. DCs have the ability to capture antigens and then present to T cells, initiating protective immune responses during infection [19]. In the present study, we examined whether vaccine *Mhp*-168 could affect DCs generated from porcine monocytes in the presence of GM-CSF and IL-4 [14]. The function of antigen presentation of DCs is related to surface molecules [20]. In this study, we demonstrated that exposure of immature BMDCs to vaccine *Mhp*-168 results in the up-regulation of MHCII and CD80/86, which stimulates immature BMDCs to develop antigen presentation functions. Generally, DCs present antigen with CD1a or MHCII as the first signal and CD80/86 as the second co-stimulatory signal to activate T cells [20]. CD1a is a member of the MHCI family which involves in the presentation of intracellular and lipid antigens [21]. Many pathogenic microorganisms, such as porcine epidemic diarrhea virus (PEDV) and Mycobacterium tuberculosis, can be presented by CD1a [22, 23]. However, surface molecule CD1a has no significant difference between vaccine *Mhp*-168 treated group and control group. This results indicate that DCs present *Mhp-168* to T cells do not via CD1a. These results indicate that vaccine *Mhp*-168 exposure causes the maturation of immature BMDCs. Up-regulating the expression of surface molecules and co-stimulatory molecules such as CD80/86, mature BMDCs present antigen information to T cells.

In addition to antigen recognition and presentation, DCs provide different cytokine microenvironments that leading differentiation of T cells into effector cells [24]. IL-10 is an anti-inflammatory cytokine, which can both impede pathogen clearance and ameliorate immunopathology [25]. Autocrine IL-10 signaling in DC can inhibit chemokine production and prevent their homing to lymph nodes, leading to the failure to recruit and induce TH1 differentiation of naïve T cells [26]. Many bacterial infections induce overexpression of IL-10, which is leading to uncontrolled pathogen growth [27]. Studies have shown that the systemic and local immune levels were not effectively activated after intranasal vaccination with the attenuated *Mhp-168* strain alone [28]. IFN-γ secretion by DCs is a crucial cytokine for the control of infectious diseases [29]. IFN-γ production were negatively regulated by IL-10 [30]. Our results demonstrate that exposure of vaccine *Mhp-168* to DCs induces significant increase in IL-10 production. The increase indicated that although *Mhp-168* exposure would induce maturation of DCs, it also promotes massive secretion of IL-10. Our study also found a down-regulation of IFN-γ produced by DCs. Overexpression of IL-10 and accompany with down-regulation of IFN-γ level may be one of the reasons why vaccination with the *Mhp-168* strain alone could not achieve productive immune effect in practical application.

Stimulation of T cell proliferation is one of the important functions of dendritic cells. We used mixed leukocyte reaction (MLR) assay to evaluate the ability of DCs to induce T cell proliferation [31]. We found that vaccine *Mhp*-168 treated BMDCs increase the proliferation of T cells both at DC/T cell ratio of 1:1 and 1:5. The stimulation level at a DC/T cell ratio of 1:1 exhibited a stronger stimulatory effect. This results indicate that primary T cells is activated by presentation of BMDCs though surface molecule MHCII and CD80/86. This results are also correlated with the highest expression of CD80/86 and MHCII in Mhp-168 treated immature BMDCs.

## Conclusions

In conclusion, this study is the first description of the interaction between vaccine *Mhp*-168 and porcine BMDCs. Our data indicates that *Mhp-168* enhances the ability of BMDCs to present and induce the proliferation of T lymphocytes. However, vaccine *Mhp-168* exposure induce significant increase in IL-10 and accompany with decrease in INF-γ expression by DCs, which indicate the possibility of immune failure in practical application with vaccine *Mhp-168* alone*.*

## Abbreviations

APC: Antigen-presenting cells
BMDCs: Bone marrow-derived DCs
DCs: Dendritic cells
EP: Porcine enzootic pneumonia
LAMPs: Lipid-associated membrane protein
MALT: Mucosa-associated lymphoid tissue
*Mhp*: *Mycoplasma hyopneumoniae*
PBMCs: Peripheral blood mononuclear cells
